# The association between circulating MicroRNA‐150 level and cholangiocarcinoma

**DOI:** 10.1002/jcla.23397

**Published:** 2020-11-07

**Authors:** Perihan El Sayed Salem, Rasha Abdelmawla Ghazala, Ahmed Mohamed El Gendi, Doaa Mokhtar Emara, Nesma Mahmoud Ahmed

**Affiliations:** ^1^ Internal Medicine Department Faculty of Medicine Alexandria University Alexandria Egypt; ^2^ Medical Biochemistry Department Faculty of Medicine Alexandria University Alexandria Egypt; ^3^ Department of Surgery Faculty of Medicine Alexandria University Alexandria Egypt; ^4^ Department of Radiodiagnosis and Intervention Radiology Faculty of Medicine Alexandria University Alexandria Egypt; ^5^ Internal Medicine Department Fever Hospital Alexandria University Alexandria Egypt

**Keywords:** cholangiocarcinoma, diagnosis of CCA, risk factors for CCA, serum CA19‐9, serum MiRNA‐150

## Abstract

Cholangiocarcinoma (CCA) is a rare tumor which requires a multimodality approach for its diagnosis. Carbohydrate antigen 19‐9 (CA19‐9) is currently the most commonly used tumor marker for CCA; nevertheless, it has certain limitations which need to be considered when using it as a tumor marker. MiRNA‐150 altered expression has been linked to the development and tumorigenesis of several cancers including CCA. This work aimed to study the serum level of CA19‐9 and miRNA‐150 expression in CCA patients and, also, to correlate their levels with tumor staging and different studied clinical and laboratory parameters. This work included 35 patients with CCA who were admitted to Hepatobiliary Unit, Alexandria Main University Hospital (Group I). Also, 35 age‐ and sex‐matched healthy subjects were included as a control group (Group II). All included subjects were submitted to measurement of serum CA19‐9 and MiRNA‐150 expression levels. Serum CA19‐9 levels showed an evident high median among CCA patients, while serum miRNA‐150 expression levels were evidently low among those patients. Moreover, combining miRNA‐150 with CA19‐9 made the accuracy of diagnosis of CCA much more reliable. Thus, miRNA‐150 can be considered as a non‐invasive, sensitive serum biomarker for the diagnosis of CCA especially when combined with CA 19‐9.

## 1 INTRODUCTION

Cholangiocarcinoma (CCA) is a rare tumor comprising approximately 3% of gastrointestinal tumors with an overall incidence of less than 2/100 000. It is the second most common primary hepatic malignancy following hepatocellular carcinoma (HCC).^1^, ^2^ CCA incidence has been increasing globally during the last decades, where its incidence increases with age.^3^ According to the anatomic location, CCA is classified into intrahepatic (IH‐CCA) and extrahepatic (EH‐CCA) where the later accounts for 80%‐90% of all cases.^4^ The commonest risk factors for CCA are primary sclerosing cholangitis (PSC),^5^ chronic liver disease (including chronic viral hepatitis B and C),^6^ and intrahepatic stones (hepatolithiasis).^7^

CCA patients remain clinically silent until the advanced stages of the disease. The common clinical feature of EH‐CCA is biliary obstruction resulting in painless jaundice, while IH‐CCA presents in most cases as an intrahepatic mass.^8^ Imaging is a cornerstone in diagnosis, including ultrasonography,^9^ computed tomography (CT),^10^ and magnetic resonance cholangiopancreatography (MRCP) which is the current gold standard for non‐invasive assessment of CCA,^11^ as well as positron emission tomography (PET)‐CT.^12^ Endoscopic retrograde cholangiopancreatography (ERCP) also has diagnostic and therapeutic roles.^13^

The most studied serum tumor marker for CCA is the carbohydrate antigen 19‐9 (CA19‐9). It is a sialylated Lewis blood group antigen targeted by the monoclonal antibody, which was described in 1979 as a tumor‐associated antigen in colorectal cancer cell line.^14^ CA19‐9 has wide variation in sensitivity (50%‐90%) and specificity (54%‐98%) in the diagnosis of CCA. Also, serum CA‐19‐9 levels depend on the Lewis phenotype, where as many as 10% of the population have been found to be Lewis negative, resulting in undetectable CA19‐9 levels; therefore, it has many limitations.^15^ It has been reported that CA19‐9 is elevated in colorectal cancers as well as nonmalignant conditions as pancreatitis, cholangitis, hepatolithiasis, and primary sclerosing cholangitis (PSC).^16^, ^17^ Thus, diagnosis of CCA still poses a challenge and is often underestimated due to the lack of a gold standard tumor marker of high specificity and sensitivity for early detection.

MiRNAs are short non‐coding RNAs of 20‐24 nucleotides that play an important role in virtually all biological pathways.^18^ MiRNAs are present not only in tissues but also can be secreted from the cells and can gain access to different body fluids, and remain highly stable upon exposure to severe conditions that induce almost immediate degradation of free RNA, such as boiling, very low or high PH, and extended storage.^19^ Therefore, circulating MiRNAs can be used as non‐invasive, sensitive biomarkers for detecting different diseases.^20^ In cancer, MiRNAs influence numerous cancer‐related processes such as metabolism, migration, proliferation, apoptosis, cell cycle control, and differentiation.^18^ Furthermore, considering that altered expression of some MiRNAs contributes to human carcinogenesis, MiRNAs have been reported to be useful as potential biomarkers for diagnosis, prognosis, and personalized therapy of different human cancers.^21^

In CCA, dysregulation of miRNA expression has not been studied extensively as in many other cancers, perhaps due to the rarity of this pathological entity. The first report on miRNA expression in human CCA was performed by Chen et al,^22^ where miRNA profiling revealed that miRNA‐18 expression was significantly up‐regulated and miRNA‐20 was down‐regulated in IH‐CCA tissues.

MiRNA‐150 is an important hematopoietic cell–specific MiRNA, mainly expressed in B cells, T cells, and natural killer cells; it plays an important role in the differentiation of some hematopoietic cell lineages particularly in lymphocyte development. MiRNA‐150 may serve as a clinically useful biomarker in the diagnosis of myeloid leukemia and may have a curative effect in hematological malignancies. It also acts as an oncogene or tumor suppressor gene in certain solid tumors depending on the cell and tumor type.^23^

MiRNA‐150 expression was up‐regulated and linked to the development and tumorigenesis of breast cancer,^24^ lung cancer,^25^ and gastric cancer.^26^ On the contrary, miRNA‐150 overexpression inhibited the malignant behavior of pancreatic cancer.^27^ Moreover, miRNA‐150 has been down‐regulated in esophageal squamous cell carcinoma^28^ and colorectal cancer.^29^

In IH‐CCA, it has been reported that plasma levels of miRNA150 were higher than healthy subjects in a small sample of patients.^30^ However, Wu et al^31^ documented that serum miRNA‐150 level was down‐regulated in CCA patients. Thus, under different cellular microenvironments, miRNA‐150 may carry out different functions. Therefore, the role of miRNA‐150 in CCA has yet to be more elucidated.

### 1.1 Aim of work

The aim of the current work was to study the serum expression level of MiRNA‐150‐5P in CCA patients and, also, to correlate its level with tumor staging and different clinical and laboratory findings of the studied patients.

## 2 SUBJECTS AND METHODS

The present study included 35 patients with CCA (randomly selected both intra‐ and extrahepatic CCA), who were admitted to Hepatobiliary Unit, Alexandria Main University Hospital (Group I). Also, 35 age‐ and sex‐matched healthy subjects were included as a control group (Group II) to obtain normal range of biochemical assays.

Diagnosis of CCA depended on CT and/or MRCP, ERCP with brush cytology (whenever needed). Also, the studied CCA patients had positive CA19‐9. Patients with other malignancies, systemic diseases (as hepatic, renal, cardiac, or respiratory diseases), sepsis, and collagenic diseases (as systemic lupus erythematosus, rheumatoid arthritis, and multiple sclerosis) were excluded. Also, all our studied CCA patients (Group I) and normal control subjects (Group II) had negative HCV Abs and HBs Ag.

All included patients were submitted to the following:

- Detailed history taking with emphasis on age, gender, yellow skin and sclera, itching, abdominal pain, anorexia and nausea, vomiting, weight loss, and change in the color of urine and stool.
- Clinical examination including both general and local abdominal examination (hepatomegaly, abdominal mass, and palpable gallbladder).
- Laboratory investigations including complete blood count (CBC), liver biochemical profile, and serum CA19‐9 level by radioimmunoassay (RIA).
- Serum expression of miRNA‐150‐5p level measured by real‐time polymerase chain reaction (RT‐PCR).

### 2.1 Method

Total serum RNA was isolated using miRNeasy Mini Kit (QIAGEN) Cat No./ID: 217004, according to the manufacturer's protocol. Briefly, 5 μL of 5 nmol/L Syn‐cel‐miR‐39 (miScriptmiRNA Mimic) was added to each sample as a spike‐in control, and then, total RNA was purified from 400 μL of sample. The miRNeasy Mini Kit contains phenol/guanidine‐based lysis of samples and silica membrane–based purification of total RNA. Samples were homogenized in Qiazol Lysis Reagent. After addition of chloroform, the homogenate was separated into aqueous and organic phases by centrifugation. RNA partitions to the upper, aqueous phase. The upper, aqueous phase was extracted, and ethanol was added to provide appropriate binding conditions for all RNA. The sample was then applied to the RNeasy Mini spin column, where the total RNA binds to the membrane and phenol and other contaminants are efficiently washed away. High‐quality RNA is then eluted in RNase‐free water. The concentration of total RNA samples from plasma was quantified by a Nanodrop 2000 (Nanodrop, USA). The range of the result was from 11.9 to 73.7 ng/μL.

The TaqMan MicroRNA Reverse Transcription (RT) Kit supplied by Applied Biosystems was used for the reverse transcription reaction. The recommended reaction volume was 20 μL. The plate was prepared, and ABI prism 7900 sequence detection system (Ambion) was used for amplification and detection by RT‐PCR. Differences in serum miRNA‐150‐5p expression were normalized to cel‐miR‐39, determined with the ΔCt method, and reported as 2^–ΔΔCt^.

### 2.2 Statistical analysis

Quantitative data of the present work were analyzed, using *F*‐test (ANOVA) and post hoc test (Scheffe) for pairwise comparison. All statistical calculations were performed using IBM SPSS software package version 20.0, where *P* < .05 was considered statistically significant.

The study was approved by the Research Ethics Committee of the Faculty of Medicine, University of Alexandria, and was conducted in accordance with the provisions of the Declaration of Helsinki and Good Clinical Practice guidelines. An informed consent was obtained from all subjects included in the study.

## 3 RESULTS

### 3.1 Demographic data

In the present study, age showed a median of 54 years and 57 years in Group I and Group II, respectively, with no statistical significant difference between both groups. As regards sex, males predominated females in both studied groups.

### 3.2 Serum CA19‐9 and MiRNA‐150‐5p expression levels

Table 1 showed comparison between both studied groups according to serum CA19‐9 and miRNA‐150‐5p expression levels. Serum CA19‐9 levels showed an evident high median among CCA (Group I) patients in comparison with normal control (Group II) subjects (413 U/mL and 20 U/mL, respectively). On the contrast, serum MiRNA‐150‐5p expression levels showed an evident low median among CCA (Group I) patients in comparison with normal control (Group II) subjects (0.04 and 1.03, respectively). An evident statistical significant difference was reported between both studied groups, where *P* < .001.

**TABLE 1:** Comparison between the two studied groups according to serum CA19‐9 and MiRNA‐150‐5p expression levels

	Group I (n = 35)	Group II (n = 35)	*U*	*P*
CA 19‐9 (U/mL)				
Min. – Max.	3.98 ‐ 3200.0	3.39 ‐ 268.0	41.0*	<.001*
Mean ± S	908.13 ± 1004	35.8 ± 82.40		
Median	413.0	20.0		
MiRNA‐150‐5p				
Min. – Max.	0.003 ‐ 1.89	0.017 ‐ 4.16	110.0*	.001*
Mean ± S	0.17 ± 0.38	1.23 ± 1.48		
Median	0.04	1.03		

Note

*U*, *P*: *U* and *P* values for Mann‐Whitney test for comparing between the two groups.

* Statistically significant at *P* ≤ .05.

### 3.3 Correlation between serum CA19‐9 and MiRNA‐150‐5p expression levels in CCA (Group I) patients

Table 2 showed negative correlation between serum CA19‐9 and miRNA‐150‐5p expression levels, where *P* = .001.

**TABLE 2:** Correlation between serum CA19‐9 and MiRNA‐150‐5p expression levels in CCA (Group I) patients

	CA19‐9 U/mL	
	*r*_s_	*P*
MiRNA‐150‐5p expression	−.533*	<.001*

Note

*r*_s_: Spearman coefficient.

* Statistically significant at *P* ≤ .05.

### 3.4 Correlation between serum MiRNA‐150‐5p expression level and different studied parameters in CCA (Group I) patients

Table 3 showed negative correlation between serum MiRNA‐150‐5p expression level and different studied parameters as well as TNM staging of CCA patients.

**TABLE 3:** Correlation between serum MiRNA‐150‐5p expression level and different studied parameters in CCA (Group I) patients

	MiRNA‐150‐5p	
	*r*	*P*
Age (y)	.149	.392
Hb (g/dL)	.128	.463
Leukocytes (×10^3^)	.194	.264
Platelets (×10^3^)	.198	.255
ALT (IU/L)	−.370*	.029*
AST (IU/L)	−.188	.279
Total bilirubin (mg/dL)	−.426*	.011*
Direct bilirubin (mg/dL)	−.366*	.031*
Alkaline phosphatase (U/L)	−.337*	.048*
GGT (U/L)	−.199	.251
Clinical presentation		
Jaundice	−.538*	.001*
Itching	−.538*	.001*
Abdominal pain	.117	.504
Anorexia and nausea	.111	.526
Vomiting	−.658*	<.001*
Weight loss	.093	.595
Change in color of urine	−.538*	.001*
Change in color of stool	−.538*	.001*
Hepatomegaly	.098	.577
TNM staging (I, II, III, IV)	−.674*	<.001*

Note

*r*: Pearson coefficient.

* Statistically significant at *P* ≤ .05.

### 3.5 Receiver operating characteristic (ROC) analysis to study the ability of serum CA19‐9, MiRNA‐150‐5p expression and combination of both for the diagnosis of CCA

**FIGURE 1:**
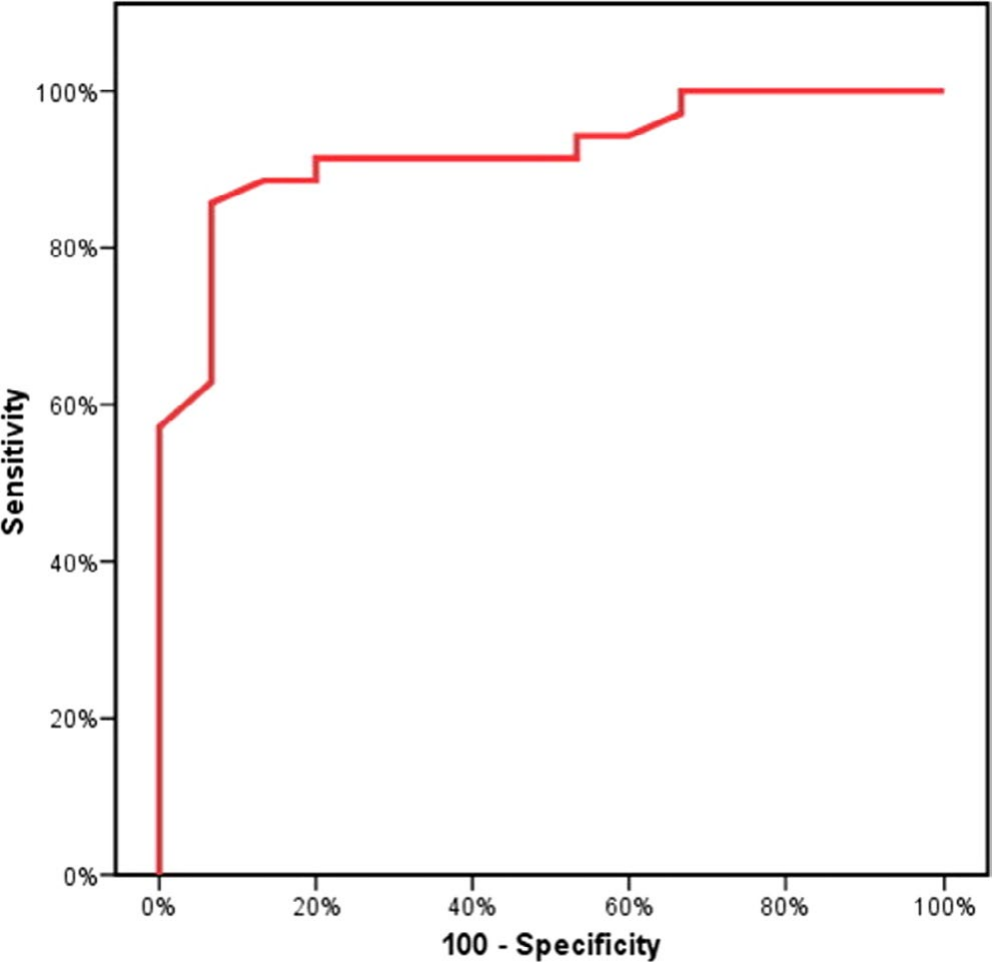
ROC analysis to study the ability of serum CA 19‐9 for the diagnosis of CCA

**FIGURE 2:**
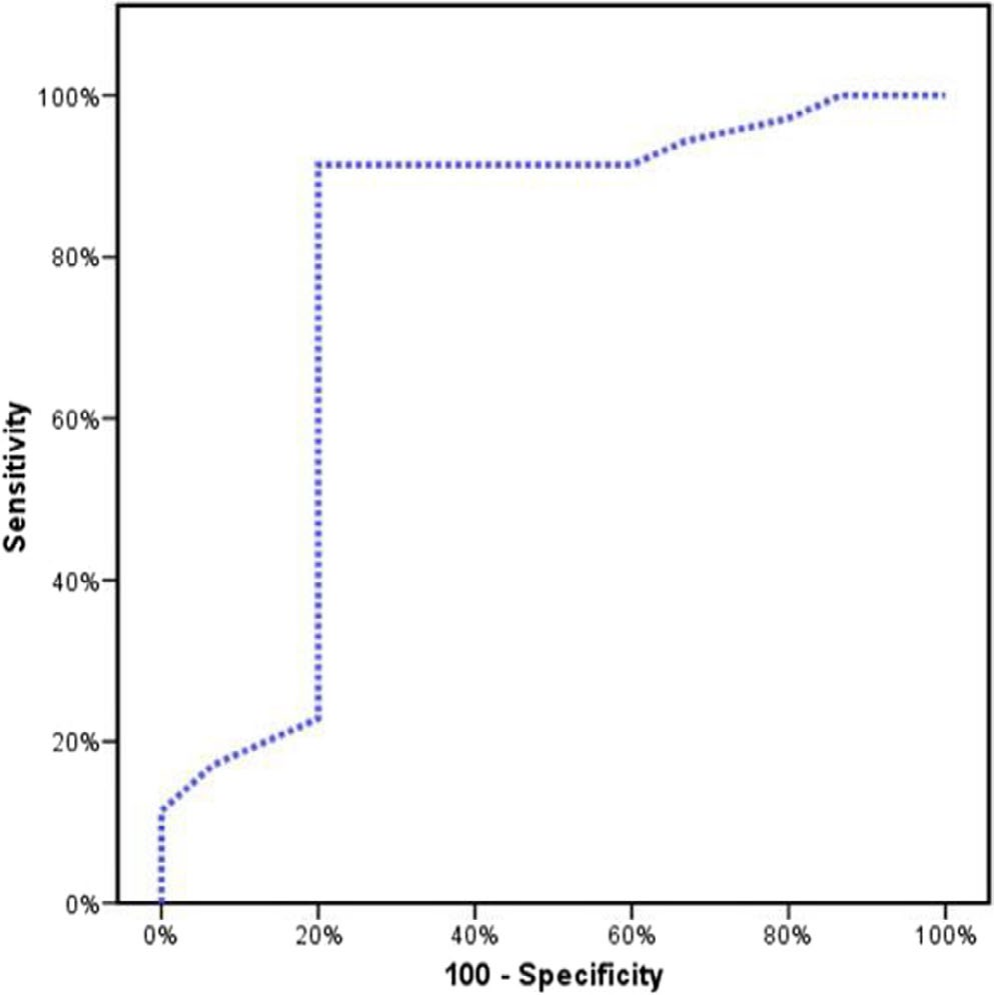
ROC analysis to study the ability of serum MicroRNA‐150‐5p expression for the diagnosis of CCA

**FIGURE 3:**
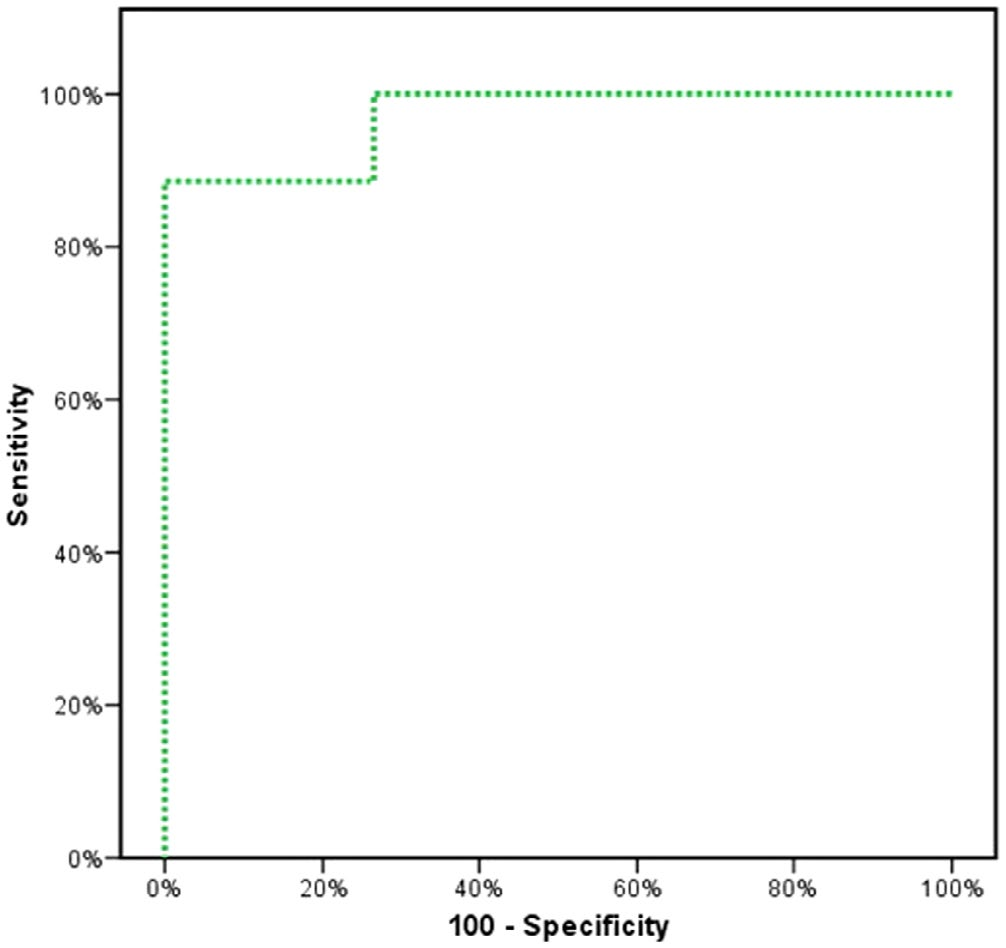
ROC analysis to study the ability of combination of both serum CA19‐9 and MiRNA‐150‐5p expression for the diagnosis of CCA

**FIGURE 4:**
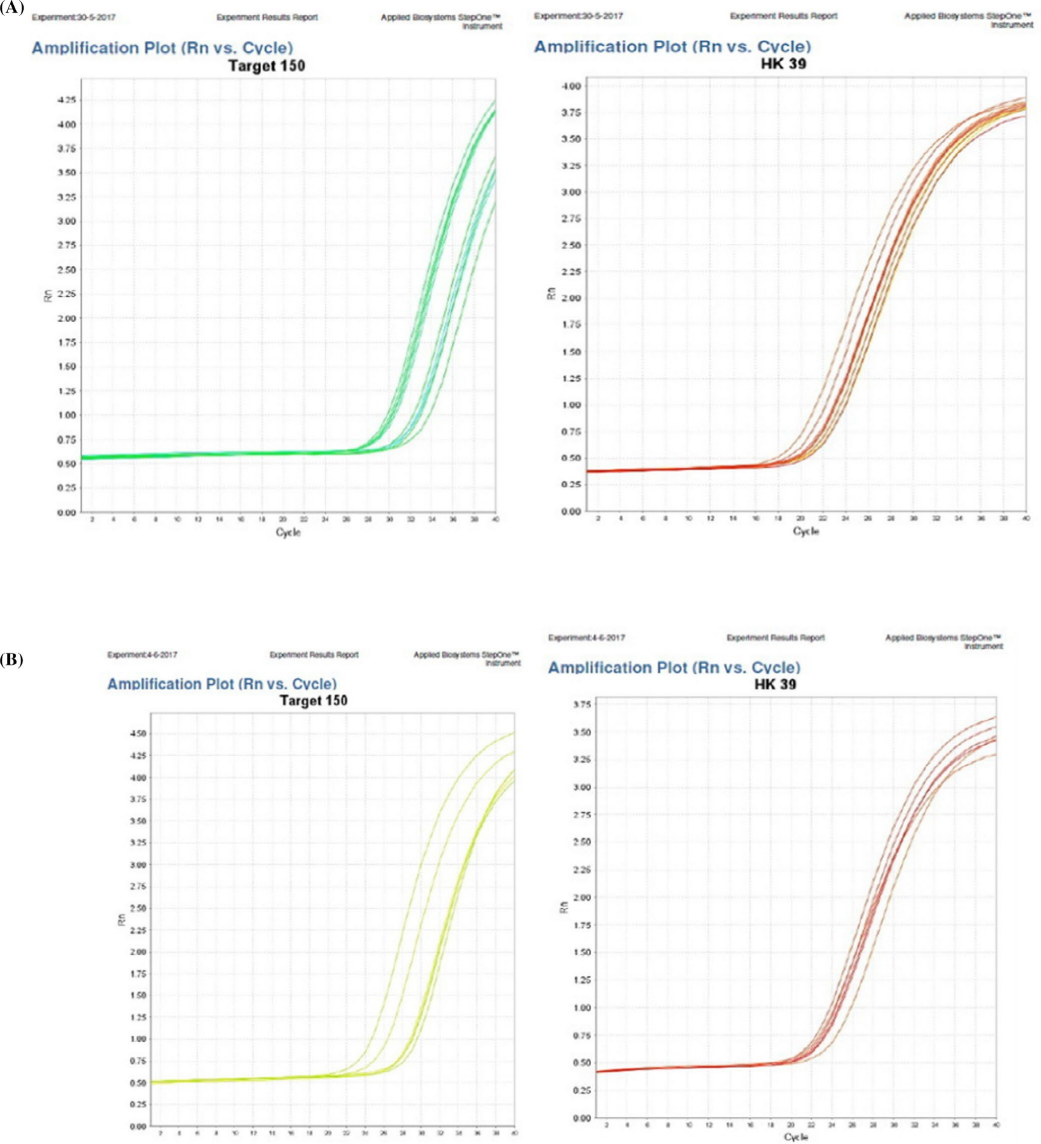
A, B, Amplification plots for the real‐time PCR. Figure (A) represents group (I), and Figure (B) represents group (II)

Figures 1, 2, 3 showed the accuracy and efficacy for the diagnosis of CCA by serum CA19‐9, serum MiRNA‐150‐5p expression, and combining both of them. Figure 4A,B showed the fluorescence signal versus cycle number for both target MiRNA‐150‐5p expression and spike‐in control cel‐mirna‐39.

## 4 DISCUSSION

CCA is a tumor with high mortality rate and is consistently asymptomatic in the early stages.^32^ Its diagnosis and staging require a multimodality approach involving laboratory, radiologic, endoscopic, and pathologic analyses. Therefore, the identification of new diagnostic and prognostic biomarkers for CCA with proper sensitivity and specificity is urgently needed.^33^

Studies have revealed that circulating miRNAs may be potential diagnostic markers in diverse diseases, especially in the field of malignant neoplasms. Hu et al^34^ reported that miRNA‐31 was significantly up‐regulated in CCA tissues, while Li et al^35^ found that miRNA‐214 was significantly lower in CCA tissues compared with normal tissues. Also, Oishi et al^36^ found that the expression of miRNA‐200 was low in CCA and was correlated negatively with overall survival and disease‐free survival.

MiRNA‐150 was found to have two contrary roles in malignant tumors. It was demonstrated that it promotes tumorigenesis in various cancers as it exerts its oncogenic function through down‐regulation of the expression of the pro‐apoptotic purinergic P2X7 receptor in epithelial cell cancer,^37^ and by targeting the pro‐apoptotic gene epidermal growth factor (EGR2) as in gastric cancer.^26^ On the contrary, other studies revealed that miRNA‐150 may act as a tumor‐suppressor miRNA, where it was found to be down‐regulated in esophageal squamous cell carcinoma,^28^ and it inhibited the growth and malignant behavior of pancreatic cancer cells by targeting mucin 4 (MUC4).^27^ Thus, whether miRNA‐150 function as oncogenes or tumor suppressors is dependent on the cell and tumor type.^38^

In the current study, serum CA19‐9 levels showed an evident high median among CCA patients which was in agreement previous studies.^39^, ^40^ On the other hand, serum miRNA‐150‐5p expression levels showed an evident low median among CCA patients which was in agreement with the finding previously observed by Wu et al,^31^ who reported that serum miRNA‐150 expression level was down‐regulated in CCA patients. They stated that miRNA‐150 was bound to an oncogene Ets including gene‐1 (ELK1), and Western blot data confirmed that miRNA‐150 suppressed ELK1 expression in CCA cell lines used in this study. These findings suggest that reduced miRNA‐150 expression in CCA patients is related to tumor development and progression. Also, Chang et al^41^ reported that miRNA‐150 was down‐regulated by c‐Myc, the later acts as one of the most common oncogenic events in human cancers, including CCA.

On the other hand, Wang et al^30^ reported contrary expression profiles of miRNA‐150 between the tumor tissues and blood samples. Authors found that miRNA‐150 expression was down‐regulated in the IH‐CCA tissues, while its plasma level was significantly higher in comparison with the controls. The difference between the extracellular and cellular miRNA‐150 profiles may be explained by the hypothesis of cellular selection mechanism of miRNA release. Based on this hypothesis, miRNA‐150 is an exocrine agent released by peritumoral non‐cancerous cells and may act as an important negative feedback regulating agent.^42^ Yet, the exact mechanism for this difference between tissue and plasma miRNA profiles is not clear and needs to be further studied.

In the present study, negative correlation between serum CA19‐9 and MiRNA‐150‐5p expression levels in CCA patients (Group I) was reported. This result was in agreement with Wu et al^31^ who stated that serum miRNA‐150 expression level was inversely associated with serum CA19‐9 level in CCA patients. This tumor suppressor action was demonstrated by Bioinformatic Kyoto Encyclopedia of Genes and Genomes (KEGG) and Gene Ontology (GO) analyses which showed that miRNA‐150 could regulate several gene pathways, including cancer pathway low expression of miRNA‐150 enhances proliferation, migration, and invasion capability of CCA cells. This action may explain the negative correlation with CA19‐9.

For better understanding of the implications of serum miRNA‐150‐5p, its expression levels were correlated with clinical and laboratory findings in the current study. There was a negative correlation between serum miRNA‐150‐5p expression level and ALT, serum bilirubin, alkaline phosphatase, jaundice, itching, vomiting, change in the color of urine and stool, and TNM staging. Our results were in accordance with Wu et al,^31^ who stated that serum miRNA‐150 level was inversely associated with pathological grades of CCA which in turn affect clinical and laboratory data.

On the other hand, Wang et al^30^ reported that there was no correlation between miRNA‐150 level and different studied laboratory parameters. They found that the miRNA‐150 level was not affected by serum albumin, total bilirubin, alanine, and aspartate aminotransferase levels as well as other clinical indices.

In the present study, CA19‐9 ability for the diagnosis of CCA at a cutoff value of 167 U/mL had a sensitivity of 85.7% and a specificity of 93.33%, and our results were in agreement with previous studies.^43^, ^44^ Moreover, serum miRNA‐150‐5p expression ability for the diagnosis of CCA had 91.43% sensitivity and 80% specificity. By combining both serum CA19‐9 and miRNA‐150‐5p expression, high accuracy and efficacy for the diagnosis of CCA were found. The sensitivity was raised to 93.33% and specificity to 96.88%, and this was in agreement with Wang et al,^30^ who reported that combining miRNA‐150 with CA19‐9 made the sensitivity and specificity for the diagnosis of CCA 80% and 100%, respectively.

## CONFLICT OF INTEREST

There is no conflict of interest in this work.

## AUTHORS CONTRIBUTIONS

Dr Perihan Salem formed the idea of the research, assessed the included patients, and wrote the paper. Dr Rasha Ghazala performed the laboratory work and revised the paper. Dr Ahmed El Gendi involved in collection of the cases and revised the paper. Dr Doaa Emara involved in imaging and revised the paper. Dr Nesma Ahmed involved in collection of the cases, performed the statistical analysis, and revised the paper.

## ETHICAL APPROVAL AND CONSENT TO PARTICIPATE

The study was approved by the Research Ethics Committee of the Faculty of Medicine, University of Alexandria, and was conducted in accordance with the provisions of the Declaration of Helsinki and Good Clinical Practice guidelines. Also, an informed consent was obtained from all subjects included in the study.

## CONSENT FOR PUBLICATION

All the contributing authors agreed for the publication in this journal.

## Data Availability

All the data related to this work are available at the corresponding author.
